# Effect of Quercetin Nanoparticles on Hepatic and Intestinal Enzymes and Stress-Related Genes in Nile Tilapia Fish Exposed to Silver Nanoparticles

**DOI:** 10.3390/biomedicines11030663

**Published:** 2023-02-22

**Authors:** Mayada R. Farag, Haitham G. Abo-Al-Ela, Mahmoud Alagawany, Mahmoud M. Azzam, Mohamed T. El-Saadony, Stefano Rea, Alessandro Di Cerbo, Doaa S. Nouh

**Affiliations:** 1 Forensic Medicine and Toxicology Department, Veterinary Medicine Faculty, Zagazig University, Zagazig 44519, Egypt; 2 Genetics and Biotechnology, Department of Aquaculture, Faculty of Fish Resources, Suez University, Suez 43518, Egypt; 3 Poultry Department, Faculty of Agriculture, Zagazig University, Zagazig 44519, Egypt; 4 Department of Animal Production College of Food & Agriculture Sciences, King Saud University, Riyadh 11451, Saudi Arabia; 5 Department of Agricultural Microbiology, Faculty of Agriculture, Zagazig University, Zagazig 44511, Egypt; 6 School of Biosciences and Veterinary Medicine, University of Camerino, 62024 Matelica, Italy; 7 Anatomy and Embryology Department, Veterinary Medicine Faculty, Zagazig University, Zagazig 44519, Egypt

**Keywords:** antioxidant capacity, intestinal bacteria, silver nanoparticles, Nile tilapia, oxidative stress, quercetin

## Abstract

Recently, nanotechnology has become an important research field involved in the improvement of animals’ productivity, including aquaculture. In this field, silver nanoparticles (AgNPs) have gained interest as antibacterial, antiviral, and antifungal agents. On the other hand, their extensive use in other fields increased natural water pollution causing hazardous effects on aquatic organisms. Quercetin is a natural polyphenolic compound of many plants and vegetables, and it acts as a potent antioxidant and therapeutic agent in biological systems. The current study investigated the potential mitigative effect of quercetin nanoparticles (QNPs) against AgNPs-induced toxicity in Nile tilapia via investigating liver function markers, hepatic antioxidant status, apoptosis, and bioaccumulation of silver residues in hepatic tissue in addition to the whole-body chemical composition, hormonal assay, intestinal enzymes activity, and gut microbiota. Fish were grouped into: control fish, fish exposed to 1.98 mg L^−1^ AgNPs, fish that received 400 mg L^−1^ QNPs, and fish that received QNPs and AgNPs at the same concentrations. All groups were exposed for 60 days. The moisture and ash contents of the AgNP group were significantly higher than those of the other groups. In contrast, the crude lipid and protein decreased in the whole body. AgNPs significantly increased serum levels of ALT, AST, total cholesterol, and triglycerides and decreased glycogen and growth hormone (*** *p* < 0.001). The liver and intestinal enzymes’ activities were significantly inhibited (*** *p* < 0.001), while the oxidative damage liver enzymes, intestinal bacterial and *Aeromonas* counts, and Ag residues in the liver were significantly increased (*** *p* < 0.001, and * *p* < 0.05). AgNPs also significantly upregulated the expression of hepatic *Hsp70*, *caspase3*, and *p53* genes (* *p* < 0.05). These findings indicate the oxidative and hepatotoxic effects of AgNPs. QNPs enhanced and restored physiological parameters and health status under normal conditions and after exposure to AgNPs.

## 1. Introduction

Thanks to nanotechnology, it has been possible to manage compounds with smaller dimensions (less than 100 nm) that facilitated their pickup by cells and made them effective in small doses. Recently, nanotechnology applications have increased in veterinary medicine [1,2,3] and particularly in aquaculture [4,5,6], ranging from nutrient and vaccine delivery to health management, water purification, pollution remediation, and fish breeding [4,7].

One of the highly demanded industrial materials are silver nanoparticles (AgNPs) [8]. AgNPs can be easily synthesized by different technologies, such as chemical, physical, and green or biological techniques [9]. Recently, AgNPs have been widely implemented in many industries, such as textiles, electronics, health care, and medical uses, because of their antimicrobial and antifungal activities [10,11]. AgNPs are used in aquaculture sectors for aquatic animal nutrition, disease control, and water treatments [12,13]. The extensive use of AgNPs in different industries increases the risk of environmental pollution as it may leak into natural water bodies during disposal, production, transportation, storage, and washing effect of the rain [14]. The aquatic ecosystem is highly sensitive to Ag^+^ ions, which dissociate from AgNPs, resulting particularly toxic [15]. The AgNPs can enter the animal bodies via endocytosis or diffusion and pass through the blood barriers affecting almost all the body organs of animals [16]. The toxicity of AgNPs has been claimed in various aquatic species, including *Daphnia magna* [17], algae [18], and fishes [19,20,21,22]. The AgNPs also altered the histological structure of the liver and gills of fish, impaired the functions of mitochondria, hampered the production of energy, induced apoptotic and oxidative damage with sublethal exposure [19,23,24,25].

Although alterations in organ histology may go unnoticed, remarkable mitochondrial changes were noticed after six months following nanoparticle exposure [26,27], suggesting long-term oxidative stress. Additionally, nanomaterials can cross the cellular membranes and, after reaching the nuclei, damage the genetic material [28], induce chromosomal aberrations and micronuclei onset in vitro and in vivo [29,30]. Exposure to AgNPs for 60 days caused high mortalities, reaching 50% (LC_50_) at 5 mg L^−1^. This was accompanied by a low-growth rate and delayed metamorphosis of the tadpole, *Polypedates maculatus* [31].

Oxidative stress and immune impairment are major obstacles in aquatic farming [32,33]. Stress induces a set of physiological responses that are compensatory or adaptive to maintain normal homeostasis [33]. Under acute or chronic stress, living organisms may lose their adaptability and balance, leading to oxidative stress, increased susceptibility to diseases, and impaired growth and reproduction [34,35,36]. The fullerene and AgNPs induced disruption of the bacterial communities (pathogenic *Vibrio* was the most prevalent genus) and antioxidant capacity of the mucus of the polychaete *Laeonereis acuta* (*Nereididae*) [37]. Furthermore, the AgNPs altered fish immunity and performance and induced metabolic disorders, inflammation, and biochemical disturbances depending on the size and concentration of nanoparticles and the exposure duration [38,39]. Therefore, it is crucial to overcome AgNPs-associated toxicity.

AgNPs-associated toxicities can be hindered by means of the application of different natural antioxidant alternatives to inhibit oxidative damage and improve fish resistance and health.

Quercetin is a promising antioxidant polyphenolic flavonoid compound of various vegetables and fruits that can protect tissues from the oxidative damaging effect of free radicals [40]. It can effectively treat a wide array of allergies, metabolic disorders, inflammations, and cardiovascular disturbances owing to its antioxidant, antiviral, antimicrobial, antidiabetic, anticancer, and antiatherosclerotic properties [41]. In Nile tilapia, the use of quercetin as a dietary supplement could improve performance, health, antioxidant mechanisms, and immune system [42]. It can also lower serum and whole body lipids, and modulate heavy metal toxicities [42]. Moreover, it showed antibacterial activity against *Pseudomonas aeruginosa* [43], *A. hydrophila* in Nile tilapia [40], and common carp (*Cyprinus carpio*) [44]. Despite these effective activities, the use of quercetin is restricted because of poor bioavailability and instability. Thus, quercetin nanoparticles (QNPs) have been developed with effective characteristics and a higher bioavailability [40]. Consequently, the current study aimed at evaluating the impact of QNPs dietary supplementation, alone or combined with AgNPs aqueous exposure, on liver function markers, hepatic antioxidant status, bioaccumulation of silver residues in hepatic tissue, whole-body chemical composition, hormonal assay, intestinal enzymes’ activity, and gut microbiota. In addition, the relative mRNA levels of some stress and apoptosis-related genes were investigated in Nile tilapia (*Oreochromis niloticus*), the predominant and most commonly cultured species in many countries, especially for intensive aquaculture.

## 2. Materials and Methods

### 2.1. AgNPs and QNPs Preparation

To obtain AgNPs, the *Bacillus subtilis* MT38 isolate was inoculated in Luria Bertani broth (LB) medium and incubated at 35 °C for 24 h. Twenty milliliters of the bacterial suspension, obtained after centrifugation at 8000 rpm for 20 min, were added to 80 mL of AgNO_3_ (3 mM) at pH 6, 30 °C, and subjected to an agitation speed of 150 rpm for 24 h. All chemicals were purchased from Sigma-Aldrich International GmbH (St. Louis, MO, USA).

To obtain QNPs, a solution with 50 mL of ethanol containing 100 mg of quercetin was prepared. The internal organic phase solutions were quickly injected into a 150 mL external aqueous solution containing the appropriate amount of polyvinyl alcohol (PVA), and then the solutions were homogenized at 20,000 rpm for 30 min. The ethanol was evaporated using a rotary vacuum evaporator at 45 °C, and the obtained material was lyophilized using a freeze dryer.

The obtained AgNPs and QNPs were characterized using UV–Vis Spectrophotometer (UV–Vis; LaxcoTM dual-beam spectrophotometer, Lake Forest, Il, USA), dynamic light scattering (DLS, Malvern Hills, Worcestershire, UK), which is a technique used to study size and charge of suspended nanoparticles, and transmission electron microscopy (TEM, JEOL 1010, Tokyo, Japan) to measure the AgNPs size in colloidal solution. Zeta potential analysis was carried out to determine the surface charge of the nanoparticles.

### 2.2. Fish and Diet Formulations

Two hundred and forty *O. niloticus* (40 ± 0.45 g body weight) were purchased from a hatchery (El-Abbassa Fish Hatchery, El-Abbassa, Al-Sharkia, Egypt) and subjected to an acclimatization period of 14 days in dechlorinated tap water in glass aquaria.

Fish were fed 3 times daily a basal diet (without AgNPs or QNPs) corresponding to a 5% of their biomass. The recommendations of the American Public Health Association regarding water quality parameters were followed [45]. The same rearing conditions were adjusted in all glass aquaria, including temperature, pH, ammonia, and dissolved oxygen, with a photoperiod of 10 h: 14 h (light: dark).

The QNPs (400 mg/kg) were mechanically mixed with the basal diet ingredients, pelletized, and left to dry at 25 °C for 24 h. The prepared diet was kept in the refrigerator at 4 °C until use. The composition of the basal diet was 32% crude protein, 45.5% fat, 42.50% fiber, 73% ash, and 518% nitrogen-free extract.

Nile tilapias were allocated into four groups (*n* = 60/group), each with four replicates (fifteen fish/replicate). Fish were kept in glass aquaria (100 × 50 × 40 cm) containing 160 L of dechlorinated tap water. The first group (control) did not receive AgNPs or QNPs in the water or the diet. The second group was fed a basal diet supplemented with 400 mg QNPs per kg diet (QNPs-supplemented group). The third group was fed a basal diet and exposed to AgNPs (1.98 mg/L; corresponding to 1/10th LC_50_). The fourth group (AgNPs/QNPs co-administered group) received QNPs and was exposed to AgNPs at the previously mentioned concentration. The daily feeding regime was performed three times at 7:00 a.m., 11:00 a.m., and 4:00 p.m. throughout the experimental period (60 days), and the amount of feed was adjusted every two weeks according to the body weight.

### 2.3. Chemical Composition of the Whole Body

On the 60th day of the experiment, five fishes were randomly selected (*n* = 5/replicate) from each group to estimate the proximate chemical composition of the whole body, represented as percentages of the wet weight [46]. The crude protein was estimated by the Kjeldahl method (Velp Scientifica, Usmate Velate, MB, Italy). The moisture was estimated by a natural convection oven (JSON-100, Gongju-City, Republic of Korea). Ash and fats were estimated by muffle furnace and Soxhlet extraction (Thermo Scientific, Greenville, NC, USA), respectively.

### 2.4. Blood and Tissue Sampling

Blood samples were collected from the caudal blood vein by sterile syringes and then placed in sterile tubes (free from anticoagulant). The samples were left to coagulate, centrifuged at 1075 g for 20 min to separate the serum, and then stored at −20 °C until physiological, biochemical, and hormonal analyses. Fish from the different groups were sacrificed by spinal cord sectioning, and the liver and whole intestine were collected. The collected organs (100 mg each) were homogenized in 10 mM phosphate/20 mM Tris-pH 7.0 using a mechanical homogenizer at 600× *g* for 3 min at 4 °C, and the supernatant was collected after centrifugation. Intestinal and liver enzymes’ activity was also analyzed.

Parts of livers were frozen until the determination of silver residues. Another set of liver tissue samples was quickly transferred to liquid nitrogen and then stored at −80 °C until RNA extraction. Other intestine samples were used for the bacterial count.

### 2.5. Serum Physiological Assays

The indices of hepatic injury, including aspartate aminotransferase (AST), alanine aminotransferase (ALT), and alkaline phosphatase (ALP), as well as total cholesterol (TC) and triglycerides (TG), were determined according to their related literature protocols [47,48,49,50,51]. Liver glycogen was determined by commercial kits (Cayman Chemical Company, Ann Arbor, MI, USA) [30].

### 2.6. Oxidative Injury Assays and Antioxidant Status

The activities of the antioxidants catalase (CAT) and superoxide dismutase (SOD), the concentration of reduced glutathione (GSH), and the oxidative injury marker malondialdehyde (MDA) were assessed in the liver tissue using a colorimetric method [52,53,54,55]. The same method was also used to monitor the protein carbonyl (PC) content in hepatic tissue (Cayman Chemical Company, Ann Arbor, MI, USA).

### 2.7. Expression of Liver Apoptosis and Stress-Related Genes

RNA was extracted from the hepatic tissue, and its integrity and concentration were checked by 1% agarose and spectrophotometry. First-strand cDNA was synthesized using a QuantiTect RT kit (Qiagen, Hilden, Germany). The primers of the tested genes (caspase3, *casp3*; heat shock protein 70, *Hsp70*; tumor suppressor protein, *p53*; the internal housekeeping gene *β-actin*) are presented in Table 1.

Real-time PCR was performed using a QuantiTect SYBR Green PCR kit (Qiagen, Hilden, Germany) and a Rotor-Gene Q apparatus. The thermocycler conditions were 95 °C for 10 min, followed by 40 cycles of 95 °C for 15 s, 60 °C for 30 s and 72 °C for 30 s. The relative expression of the studied genes was analyzed using the 2^−ΔΔCt^ equation [60].

### 2.8. Intestinal Enzyme Activities

The intestinal lipase and α-amylase activities were estimated with a fast colorimetric kit (Spectrum Diagnostic Co., Cairo, Egypt) [61,62], according to the manufacturer’s directives. The intestinal protease activity was estimated according to the method proposed by Bezerra et al. [63].

### 2.9. Hormonal Assay

Fish GH, T3, T4, and glucagon were estimated in the serum using ELISA kits (catalog numbers MBS701414, MBS2700145, MBS701162, MBS034316, respectively; MyBioSource, San Diego, CA, USA).

### 2.10. Determination of Aeromonas Counts and Total Intestinal Bacteria

Intestine samples were taken from 5 fish/group to enumerate *Aeromonas* and total bacteria. The samples were homogenized in sterile saline peptone water (8.5 gL^−1^ NaCl and 1 gL^−1^ peptone), followed by serial dilution up to 10^7^. The total bacteria and *Aeromonas* were counted after incubation at 37 °C for 24 h on plate count agar [64] and agar medium [65], respectively.

### 2.11. Determination of Silver Residues

The liver samples were exposed to digestion by acids [66]. One gram of each sample was transported to a screw-capped glass bottle and exposed to a 4 mL digestion solution of nitric and perchloric acid (1:1). The samples were left at room temperature for 24 h for an initial digestion and then heated for 2 h at 110 °C. After that, the samples were cooled, and deionized water was added. Then, the solutions were warmed in a water bath for 1 h to eliminate nitrous gases. The digestion products were filtered, and deionized water was added up to 25 mL. Silver residues were determined by flame atomic absorption spectrophotometer (FAAS).

### 2.12. Statistical Analysis

The obtained data were statistically analyzed by SPSS (version 16.0, SPSS Inc., Chicago, IL, USA). All data are presented as means ± standard deviation. One-way analysis of variance (ANOVA) with Tukey’s multiple comparison *post hoc* test was applied to compare means among groups (* *p* < 0.05).

## 3. Results

### 3.1. AgNPs and QNPs Characterization (Surface Chemistry)

**Figure 1:**
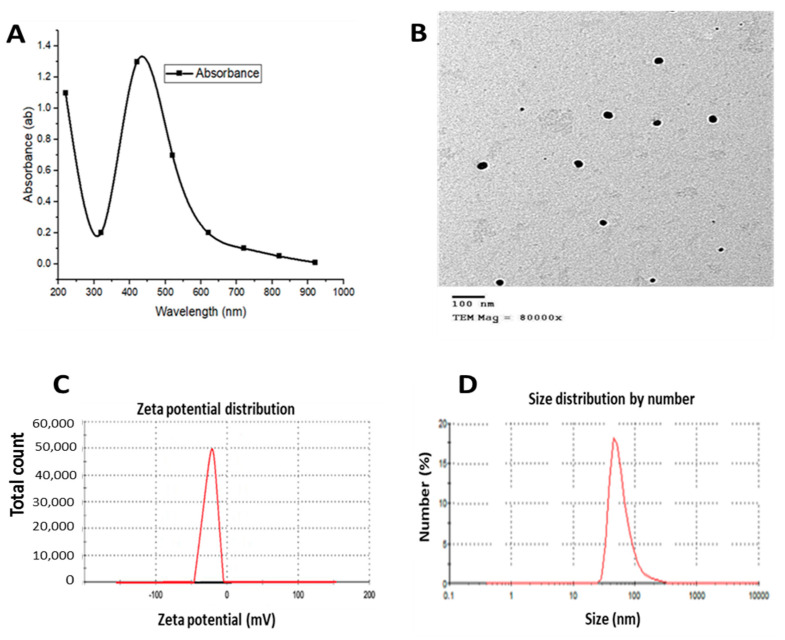
Characterization of AgNPs. (**A**) UV–Vis spectroscopy shows the maximum peak at 420 nm, (**B**) TEM analysis reveals a spherical shape 30–60 nm average size, (**C**) the zeta potential analysis shows a −22 mV net surface charge, (**D**) the DLS analysis shows a hydrodynamic size of 59 nm.

The results of the characterization of AgNPs are presented in Figure 1. UV–Vis spectroscopy results showed the maximum peak at 420 nm. TEM analysis revealed a spherical shape with an average size of 30–60 nm and a net surface charge of −22 mV. According to the DLS analysis, the exact size was 59 nm.

**Figure 2:**
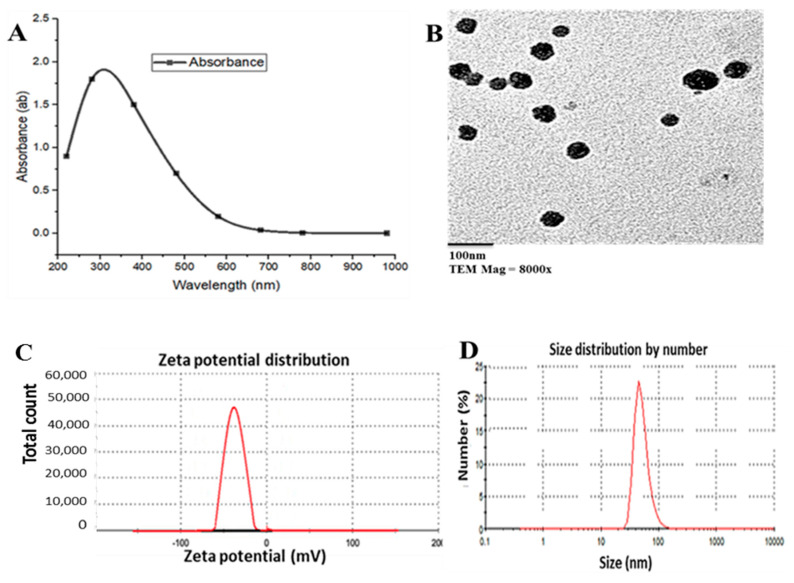
Characterization of QNPs. (**A**) UV–Vis spectroscopy results show the maximum peak at 310 nm, (**B**) TEM analysis reveals a spherical shape with an average size of 45–65 nm, (**C**) zeta potential analysis shows a net surface charge of −23 mV, (**D**) the DLS analysis shows a hydrodynamic size of 77 nm.

Regarding the QNPs, TEM analysis revealed a spherical shape absorbing UV at 310 nm, an average size of 45–65 nm, and a net surface charge of −23 mV. DLS analysis showed an exact size of 77 nm (Figure 2).

### 3.2. Whole-Body Chemical Composition

The moisture percent of fish that received AgNPs was significantly higher than that of the other groups by approximately 3.5% (Table 2). The same trend was also observed in the ash, which recorded an increase of 1.8% compared to the QNPs and control groups. Fish that received AgNPs and QNPs showed increased ash percentages; however, these increases were nonsignificant and lower than those in the AgNP group.

The crude lipid percentage showed significant changes among the treated groups; the lowest and highest values were observed in the AgNPs and control groups. The crude lipid percentage of groups that received AgNPs + QNPs or AgNPs was around 5%. AgNPs markedly reduced the crude protein percentage, and such a decrease remained significantly lower than those of the QNPs and control groups.

### 3.3. Serum Physiological Assays

AgNPs notably increased serum levels of ALT and AST, with values double to triple those of the control; while QNPs significantly reduced these close to those of the control (Table 3). The glycogen level was significantly low in the AgNP group; however, this effect was rescued in the AgNPs + QNPs group.

QNPs significantly reduced the levels of TG and TC in serum levels in the QNPs group and kept them at lower values than those of the control.

### 3.4. Antioxidant Status and Oxidative Injury Assays

The activities of CAT, SOD, and GSH were significantly inhibited in the liver of the AgNP group (Table 4). Notably, GSH recorded a very low activity in the AgNP group, which reached a third of the values of the control group. MDA and PC levels were increased in the liver in response to AgNPs exposure. QNPs improved the negative effect of AgNPs on the activities of SOD, CAT, and GSH and, to a reasonable extent, increased the activities of MDA and PC in the liver.

### 3.5. Expression of Apoptosis and Stress-Related Genes

**Figure 3:**
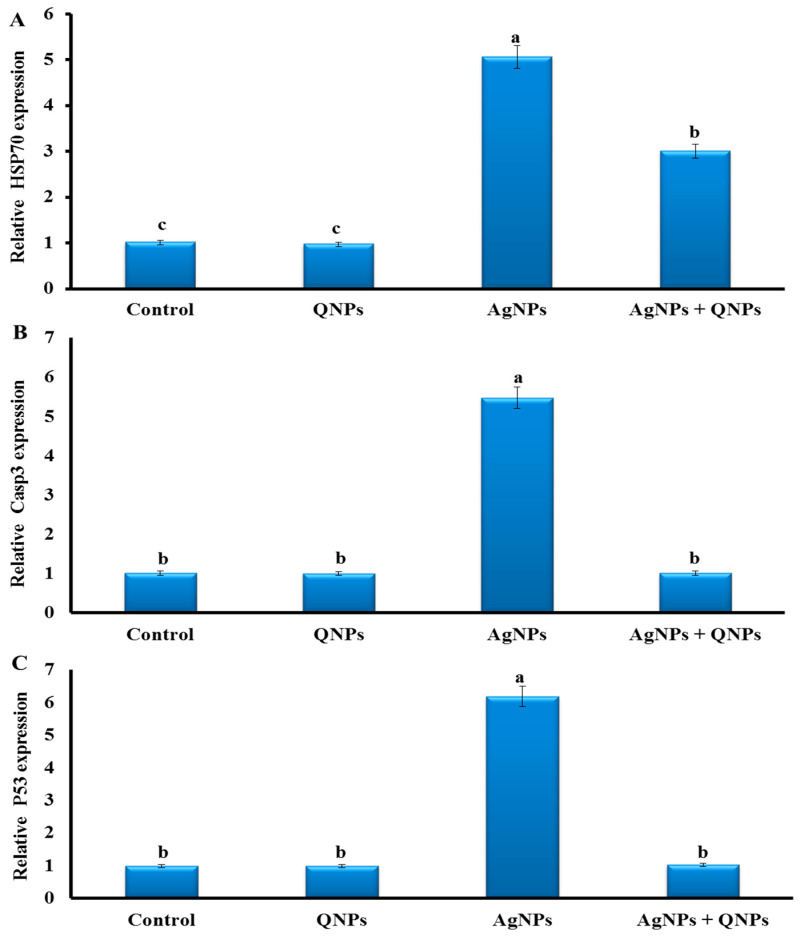
Expression of the stress-related genes (**A**) *Hsp70*, (**B**) *casp3*, and (**C**) *p53* in the livers of the studied groups: Control group (basal diet), QNPs group (QNPs at a concentration of 400 mg kg^−1^ diet), AgNP group (AgNPs at a level of 1.98 mg L^−1^), and AgNP + QNP group (QNPs and AgNPs). Data were normalized using the reference gene *β-actin*. Values are presented as mean ± SEM. Values with a common superscript letter (a, b, c) significantly differ (*p* < 0.05).

The expression of the hepatic *Hsp70*, *casp3,* and *p53* genes was significantly upregulated in the AgNP group, with values between five- and six-fold increases (Figure 3). The expression of these genes was unaffected by QNPs treatment. Interestingly, the expression levels of these genes returned to the normal range in the AgNPs + QNPs group, except for *Hsp70*, which decreased by two-fold and remained at higher levels than the control.

### 3.6. Intestinal Enzyme Activity

QNPs increased intestinal enzyme activities (i.e., amylase, lipase, and protease) (Table 5). QNPs preserved much of the reduced intestinal enzyme activities resulting from AgNPs challenge. QNPs showed a marked effect on intestinal lipase activity in the QNP and AgNP + QNP groups.

### 3.7. Hormonal Assay

The GH, T3, T4, and glucagon levels were lowered in the AgNP group; however, QNPs kept them at normal levels in the AgNP + QNP group (Table 6). The changes in GH were statistically significant, while those in T3, T4, and glucagon were not significant.

### 3.8. Total Intestinal Bacteria and Aeromonas Counts

**Figure 4:**
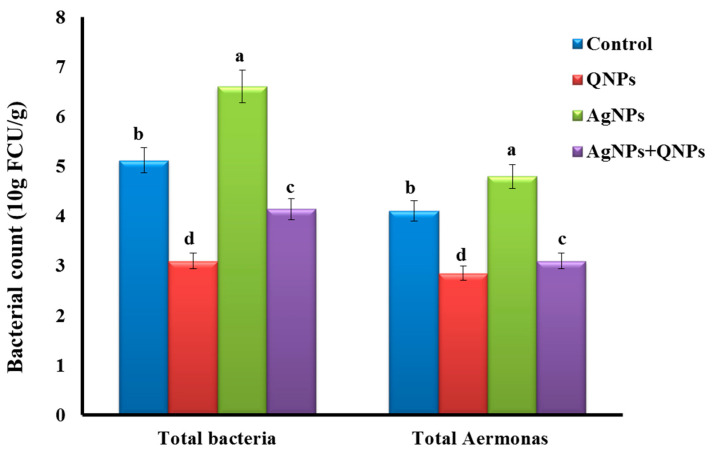
Total bacteria and *Aeromonas* counts in the fish intestine. Control group (basal diet), QNPs group (QNPs at a concentration of 400 mg kg^−1^ diet), AgNP group (AgNPs at a level of 1.98 mg L^−1^), and AgNP + QNP group (QNPs and AgNPs). Values are presented as mean ± SEM. Values with a common superscript letter (a, b, c, d) significantly differ (*p* < 0.05).

Notably, AgNPs markedly increased the total intestinal bacteria and *Aeromonas* count in the AgNP group (Figure 4). However, QNPs significantly decreased the total intestinal bacterial and *Aeromonas* counts in the QNP and AgNP + QNP groups compared to the control and AgNP groups.

### 3.9. Silver Residues

**Figure 5:**
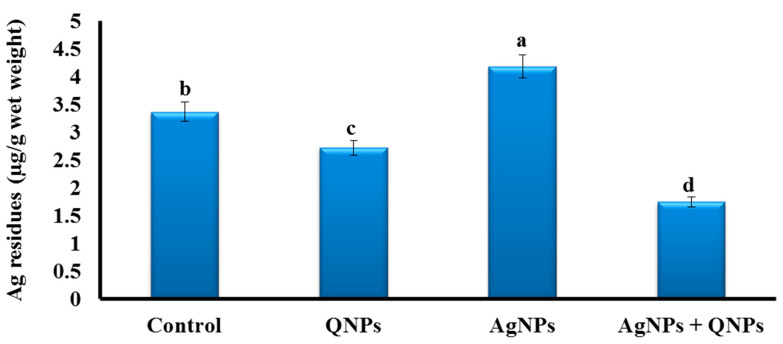
Bioaccumulation of silver residues in the liver of the studied groups. Control group (basal diet), QNPs group (QNPs at a concentration of 400 mg kg^−1^ diet), AgNP group (AgNPs at a level of 1.98 mg L^−1^), and AgNP + QNP group (QNPs and AgNPs). Values are presented as mean ± SEM. Values with a common superscript letter (a, b, c, d) significantly differ (*p* < 0.05).

The highest level of silver residues was detected in the liver of the AgNP group compared to other groups (Figure 5). QNPs lowered the silver residues in the liver.

## 4. Discussion

The rapid expansion in the applications of engineered nanomaterials showed environmental impacts that are gaining greater and greater attention, associated with their novel advantages and potential hazards to living creatures. The AgNPs’ toxicity was investigated and found to be dependent on the shape, coating material, size, dose, duration of exposure, and species differences [9,67].

Characterization of AgNPs showed a spherical shape with an average size of 30–60 nm under TEM. UV–Vis spectroscopy showed the maximum peak at 420 nm with −22 mV net surface charge by zeta potential analysis, while the DLS analysis showed the hydrodynamic size of 59 nm. AgNPs have been already characterized for size and dispersity using UV–Vis spectroscopy and TEM, showing a peak at 431 nm with the size distribution ranging from 60 to 80 nm, respectively [68]. Shaluei et al. (2013) reported an average nanoparticle size of 61 nm [69]. The morphological characteristics of AgNPs by TEM showed mono-dispersed, roughly spherical with average sizes from 80 to 90 nm without any agglomeration. The spherical configuration of AgNPs under TEM was also observed by Srinonate et al. [70]. The data of DLS analysis showed that the Z-average was 32.20 nm [71]. Sibiya et al. (2022) reported a typical high-pitched peak of absorbance recorded on UV–Vis spectrophotometer at 450 nm due to the absorption of AgNPs surface plasmon resonance which confirmed the reduction of silver nitrate [72]. The same authors examined the size, shape, and morphology of AgNPs using TEM proving that AgNPs were globular in shape. other studies reported spherical and scattered smaller-sized AgNPs with approximately 20 nm in size [73,74]. The variations among previous studies and the present one might be ascribed to the different method of AgNPs synthesis.

AgNPs significantly increased serum ALT and AST, with double to triple values compared to the control. Indeed, elevated serum ALT and AST levels are considered as liver injury and stress markers [75,76]. Indeed, both regulate the transamination process, particularly during stress, to fulfill the increased energy requirement of the body [77], and modulate the metabolism of carbohydrates and proteins [78,79,80]. Thus, the activities of ALT, AST, but also ALP are highly indicated to measure the fish toxicity and recovery pattern [81].

In accordance, the ALT and ALP activities in common carp and ALP and acid phosphatase in *Labeo rohita* were significantly enhanced following exposure to AgNPs [22,82]. This increased activity could be ascribed to disruption of hepatocyte membranes and leakage of such enzymes from the hepatic cells into the bloodstream [25]. At the same time, the liver is an early target of detoxification and accumulation of various toxic substances [21]. The exposure to AgNPs enhanced the reactive oxygen species (ROS) production in the hepatoma cell line derived from fish [83], which is also confirmed by the increased MDA and PC levels in our findings. This oxidative stress could disrupt the function of mitochondria and lead to toxic effects by decreasing the integrity of the cell membrane and oxidizing the constituents of the cell [84].

ALT serum levels have been shown to be associated with liver fat [85,86]. In fish and mammals, de novo lipogenesis plays a crucial role in glucose homeostasis, in which lipogenic enzyme activities are modulated by dietary carbohydrate intake [87,88,89] and thus modulate glycogen levels [90].

Since the liver appeared to be targeted by AgNPs, hepatic glucagon signaling seemed to be inhibited, leading to decreased serum glucagon, as seen in the present study. Glucagon receptor signaling is linked to the metabolism of lipids [91] and amino acids [92]. Blockade of the glucagon receptor decreased hepatic amino acid catabolism with increased serum amino acids in animal models, including zebrafish [92,93,94]. Knockdown of the glucagon receptor upregulated the expression of hepatic lipogenic genes, increased hepatic lipid contents, and enhanced de novo lipid synthesis [95]. Glucagon inhibits hepatic de novo lipogenesis by the cyclic AMP-responsive element-binding protein H-insulin-induced gene-2a signaling pathway [96]. In the AgNP group, the whole body’s crude lipid and protein percentages were lower than the control. Accordingly, AgNPs may modulate glucagon receptor signaling. Although QNPs decreased the crude lipid content compared to the control (i.e., by approximately 1%), they beneficially increased the protein content in the whole body. QNPs also increased the lowered levels of the crude lipid and protein percentages caused by AgNPs.

Glucagon is secreted to regulate blood glucose levels and is strongly suggested to promote ureagenesis to regulate amino acid metabolism [97,98,99].

Hepatic knockdown of the glucagon receptor increased total plasma cholesterol and increased triglycerides [95]. Quercetin inhibited the increases in plasma cholesterol and protected pancreatic β-cells from oxidative stress, mitochondrial dysfunction (e.g., decreased ATP levels), and lipid peroxidation induced by high cholesterol treatment in vivo and in vitro [100]. Quercetin facilitates cholesterol excretion and helps protect cells from excessive accumulation of cholesterol by enhancing reverse cholesterol transport through the upregulation of related protein expression [101]. Typically, our findings indicated that QNPs decreased the TC and TG in the QNP and AgNP + QNP groups to lower levels than in the control group.

AgNPs have a direct effect on SOD, CAT, GSH, MDA, and glutathione peroxidase (GPx), which can change the antioxidant capacity [102]; they also initiate the production of ROS [103]. These enzymes are responsible for the detoxification of ROS and normal homeostasis maintenance. If the antioxidant system cannot maintain safe levels of ROS, oxidative stress occurs, and cellular damage may develop [32]. Mansour et al. (2021) showed a depletion of the activities of antioxidant enzymes and significant MDA production, as an indicator of ROS, in fish exposed to AgNPs at high levels. Similarly, *O. niloticus* and *Tilapia zillii* exposed to AgNPs (4 mg/L) showed reduced gene expressions and activity of antioxidant enzymes and enhanced levels of MDA in the brain of treated fish [16]. The SOD, CAT, and GST activities were significantly reduced in different organs of *Labeo rohita* following the exposure to increasing AgNPs concentrations [82]. 

AgNPs from wastewater led to oxidative damage and reduction of SOD activity in rainbow trout [104]. Moreover, exposure of common carp (*C. carpio*) to AgNPs (12.5% of LC_50_) increased the activity of CAT and SOD while exposure to 25% and 50% of LC_50_ showed opposite effects [22].

Sibiya et al., (2022) showed that AgNPs induced oxidative stress by increasing the activity of PC and lipid peroxidation in the gills, and altered the antioxidants such as GPx, glutathione-S-transferase (GST), CAT, SOD and GSH in *O. mossambicus* [72]. Furthermore, the AgNPs can interfere with the synthesis of antioxidant enzymes [105].

Therefore, the decrease in antioxidant enzyme activity observed in the present study could be attributed to the depression of antioxidant genes expression and enzyme synthesis process leading to the weakening of the cell antioxidant capacity [84,106]. The mechanism behind this weakening is the nanoparticles’ metallic nature, and the existence of ionic forms of transition metals that encourage ROS production leading to oxidative stress [107]. Our results showed that QNPs have effective antioxidant activities against the oxidative damage induced by AgNPs in the liver. Earlier reports indicated that quercetin markedly protected against the decreased activities of SOD and GPx induced by high cholesterol supplementation in animal models and in vivo [100]. In zebrafish, nano-encapsulated quercetin maintained redox status after exposure to AgNPs [108]. QNPs had moderate but effective preservation of the MDA content; however, they could not restore the activity of MDA to physiological levels. This finding could be explained by the variable resistance of the antioxidant activities toward AgNPs, in which MDA showed less resistance to AgNPs and Ag^+^ [102]. However, the other antioxidant enzymes had variable resistance against AgNPs and Ag^+^, and SOD showed stronger resistance to both forms of silver [102].

Quercetin protects against inflammatory/oxidative stress responses by modulating 5’adenosine monophosphate-activated protein kinase (AMPK)/sirtuin 1 (SIRT1)/nuclear factor kappa B (NF-κB) signaling, which upregulates the expression of SIRT1 and downregulates NF-κB [109,110]. The induction of NF-κB prompts the expression of related stress genes (e.g., heat shock proteins) [111]. AgNPs upregulated *Hsp70* and *p53* (cell cycle checkpoint proteins that control cell division and apoptosis, respectively), inhibited the antioxidant GSH, and enhanced MDA and the apoptosis markers *casp3* and *casp9*, indicating induced oxidative stress, nucleic acid damage, and apoptosis in the genetic model *Drosophila melanogaster* [112].

According to our results, AgNPs upregulated the expression of *Hsp70*, *p53*, and *casp3.* AgNPs were already shown to induce inflammatory response, oxidative stress and *Hsp70* stress gene expression upregulation in Nile tilapia [8]. AgNPs toxicity also induced *p53* expression in the liver tissue of adult zebrafish [74]. Moreover, p53 activation in response to DNA damage can lead to cell cycle arrest or apoptosis preventing cell proliferation [113,114]. However, this action was rescued by QNPs, with a lesser effect on *Hsp70*, which showed an antiapoptotic effect by suppressing *casp3* and releasing cytochrome c [100].

In the present study, intestinal enzyme activities (i.e., amylase, lipase, and protease) and GH, T3, and T4 were checked to assess the physiological status of the digestion process and growth. The results of the exposure to AgNPs are consistent with the disrupted growth performance observed after increasing the concentration of AgNPs in the Nile tilapia [71].

The findings revealed improved intestinal enzyme activities by QNPs in both the QNP and AgNP + QNP groups. Importantly, QNPs exhibited a pronounced effect on intestinal enzyme activities in the QNP group. This could be attributed to the protection of quercetin against intestinal oxidative damage and the maintenance of intestinal barrier function [115,116]. Furthermore, total intestinal bacteria and *Aeromonas* counts were unexpectedly increased in the AgNP group owing to silver antibacterial activity [103]. A possible explanation of this observation is that a high concentration of AgNPs negatively modulated the intestinal microbiota and increased harmful bacteria such as *Aeromonas*. Earlier studies support this hypothesis, showing that AgNPs caused gut dysbiosis in animal models, including fish [117,118,119,120].

Silver residues were highly detected in the liver, and the current findings indicate a primitive role of the liver in the detoxification of silver and AgNP-induced liver cell injury. Similar results were observed in *Clarias gariepinus* and Indian major carp *Labeo rohita*, in which AgNPs were highly detected in the liver even after 15 days of recovery [121,122].

Additionally, AgNPs were massively accumulated in the liver of common carp (*C. carpio*) [22]. Further, silver residues showed the highest levels in gills compared to other tissues in common carp and African catfish (*C. gariepinus*) [22,123]. Such variations may depend on species-specific differences or variable experimental conditions. For instance, 15 days of exposure to silver led to its considerable accumulation in the liver of *C. gariepinus* [121,122]. Treatment of the freshwater rainbow trout with AgNPs resulted in the accumulation of great quantities of silver in the liver, intestine, muscles, and gills [124]. Moreover, 100 μg/L of AgNPs or AgNO_3_, individually or combined with 10 mg/L of humic acids, bioaccumulated Ag in gills and altered the antioxidant status of *Piaractus mesopotamicus* [125]. The ability of freshwater fish to accumulate AgNPs and AgNO_3_ may impair their biochemical and physiological parameters [126].

## 5. Conclusions

In conclusion, our findings showed that AgNPs (1.98 mg/L) have a deleterious effect on the physiological status and antioxidant system of Nile tilapia. They markedly increased serum levels of ALT, AST, TC, and TG. SOD, CAT, and GSH were significantly inhibited in the liver, and the expression of hepatic stress-related genes was upregulated after exposure to AgNPs. In addition, the intestinal enzyme activities and bacterial counts were disrupted. This indicates a hepatotoxic effect of AgNPs. QNPs showed promising protective action against the impact of AgNPs. Additionally, QNPs exhibited beneficial effects in enhancing the physiological and health status and growth parameters of Nile tilapia when used under normal conditions.

## 6. Limitations and Future Perspectives

Various reports documented the possible toxic effects of AgNPs in vitro and in vivo. Therefore, investigating the mechanism of interaction between biological cells and AgNPs to better understand their potential risks as antibacterial agents seems to become a significant issue. Moreover, transforming some natural polyphenolic compounds, such as quercetin, into QNPs may provide better physical insights, thus enhancing their pharmaceutical efficacy.

## Figures and Tables

**Table 1:** Primer sequences (forward and reverse) used for expression analysis.

Gene Symbol	Sequence (5’-3’)	Gene Name	Accession Number	Reference
*p53*	F: GCATGTGGCTGATGTTGTTC R: GCAGGATGGTGGTCATCTCT	Tumor suppressor protein	FJ233106.1	Farag, et al. [56]
*casp3*	F: GGCTCTTCGTCTGCTTCTGT R: GGGAAATCGAGGCGGTATCT	Caspase3	GQ421464.1	Standen, et al. [57]
*Hsp70*	F- CTCCACCCGAATCCCCAAAA R: TCGATACCCAGGGACAGAGG	Heat shock protein 70	EU816596.1	Hassan, et al. [58]
*β-actin*	F: AGCAAGCAGGAGTACGATGAG R: TGTGTGGTGTGTGGTTGTTTTG	Beta-actin	XM-003455949.2	Pang, et al. [59]

**Table 2:** Effect of QNPs on whole-body composition (% wet weight basis) in AgNPs-induced toxicity in Nile tilapia.

	Control	QNPs	AgNPs	AgNPs + QNPs	*p*-Value
Moisture (%)	76.17 ± 0.38 ^b^	75.81 ± 0.45 ^b^	79.48 ± 0.14 ^a^	75.93 ± 0.43 ^b^	<0.001
Ash (%)	4.41 ± 0.23	4.34 ± 0.15	6.25 ± 0.24	5.14 ± 0.17	0.814
Crude lipid (%)	6.19 ± 0.03 ^a^	5.07 ± 0.03 ^b^	4.14 ± 0.04 ^d^	4.84 ± 0.04 ^c^	<0.05
Crude protein (%)	14.02 ± 0.45 ^a^	14.44 ± 0.17 ^a^	11.30 ± 0.07 ^c^	12.58 ± 0.16 ^b^	<0.001

Values are presented as mean ± SEM. Values with common superscript letters (a, b, c, d) significantly differ (*p* < 0.05).

**Table 3:** Effect of QNPs on blood parameters in AgNPs-induced toxicity in Nile tilapia.

	Control	QNP	AgNPs	AgNPs + QNPs	*p*-Value
ALT (IU L^−1^)	26.98 ± 0.53 ^c^	24.28 ± 0.12 ^c^	92.37 ± 3.15 ^a^	33.70 ± 1.23 ^b^	<0.001
AST (IU L^−1^)	53.31 ± 0.57 ^b^	40.85 ± 0.64 ^c^	151.66 ± 0.61 ^a^	53.84 ± 0.92 ^b^	<0.001
Glycogen (pg mL^−1^)	74.42 ± 0.35 ^a^	73.27 ± 0.87 ^a^	44.28 ± 0.49 ^b^	72.67 ± 1.34 ^a^	<0.001
TC (mg dL^−1^)	181.07 ± 9.60 ^b^	154.76 ± 3.11 ^c^	216.40 ± 2.39 ^a^	175.20 ± 3.42 ^ab^	<0.001
TG (mg dL^−1^)	99.79 ± 6.89 ^b^	65.92 ± 5.66 ^c^	121.44 ± 1.88 ^a^	89.51 ± 0.92 ^b^	<0.001

Values are presented as mean ± SEM. Values with common superscript letters (a, b, c) significantly differ (*p* < 0.001).

**Table 4:** Effect of QNPs on oxidative stress in AgNPs-induced toxicity in liver of Nile tilapia.

	Control	QNPs	AgNPs	AgNPs + QNPs	*p*-Value
SOD (U g^−1^ tissue)	5.48 ± 0.20 ^a^	5.67 ± 0.07 ^a^	3.13 ± 0.05 ^c^	4.77 ± 0.15 ^b^	<0.001
CAT (U g^−1^ tissue)	4.45 ± 0.07 ^a^	4.12 ± 0.01 ^a^	3.05 ± 0.05 ^b^	3.16 ± 0.32 ^b^	<0.001
GSH (nmol g^−1^ tissue)	2.77 ± 0.05 ^a^	3.26 ± 0.26 ^a^	0.82 ± 0.02 ^b^	2.74 ± 0.05 ^a^	<0.001
MDA (nmol g^−1^ tissue)	14.57 ± 0.17 ^b^	13.19 ± 0.09 ^c^	18.46 ± 0.32 ^a^	14.58 ± 0.17 ^b^	<0.001
PC (nmol g^−1^ tissue)	4.23 ± 0.01 ^c^	4.19 ± 0.39 ^c^	7.75 ± 0.37 ^a^	5.77 ± 0.38 ^b^	<0.001

Values are presented as mean ± SEM. Values with common superscript letters (a, b, c) significantly differ (*p* < 0.001).

**Table 5:** Effect of QNPs on intestinal enzyme activity in AgNPs-induced toxicity in Nile tilapia.

	Control	QNPs	AgNPs	AgNPs + QNPs	*p*-Value
Amylase (DU)	1.16 ± 0.17 ^a^	1.37 ± 0.01 ^a^	0.34 ± 0.02 ^c^	0.80 ± 0.06 ^b^	<0.001
Lipase (FCCFIP)	52.54 ± 0.91 ^b^	85.31 ± 2.61 ^a^	27.16 ± 1.16 ^c^	51.41 ± 0.83 ^b^	<0.001
Protease (HUT)	1.52 ± 0.01 ^b^	3.49 ± 0.03 ^a^	0.44 ± 0.03 ^d^	0.93 ± 0.01 ^c^	<0.001

Values are presented as mean ± SEM. Values with common superscript letters (a, b, c, d) significantly differ (*p* < 0.001).

**Table 6:** Effect of QNPs on hormones in AgNPs-induced toxicity in Nile tilapia.

	Control	QNPs	AgNPs	AgNPs + QNPs	*p*-Value
GH (pg mL^−1^)	560.07 ± 5.18 ^a^	560.17 ± 1.64 ^a^	344.09 ± 6.06 ^b^	539 ± 8.02 ^a^	<0.001
T3 (pg mL^−1^)	302.00 ± 13.58	301.33 ± 12.25	241.10 ± 28.12	301.67 ± 29.07	0.214
T4 (ng mL^−1^)	133.27 ± 2.58	94.00 ± 4.26	77.97 ± 3.77	134.48 ± 2.50	0.199
Glucagon (pg mL^−1^)	4.59 ± 0.05	4.58 ± 0.04	4.59 ± 0.05	4.58 ± 0.8	0.999

Values are presented as mean ± SEM. Values with common superscript letters (a, b) significantly differ (*p* < 0.001).

## Data Availability

The data presented in this study are available on request to the corresponding authors.
